# The correlation of retinal neurodegeneration and brain degeneration in patients with Alzheimer’s disease using optical coherence tomography angiography and MRI

**DOI:** 10.3389/fnagi.2023.1089188

**Published:** 2023-04-12

**Authors:** Bingying Zhao, Yibing Yan, Xingqi Wu, Zhi Geng, Yue Wu, Guixian Xiao, Lu Wang, Shanshan Zhou, Ling Wei, Kai Wang, Rongfeng Liao

**Affiliations:** ^1^Department of Ophthalmology, The First Affiliated Hospital of Anhui Medical University, Hefei, Anhui, China; ^2^Department of Neurology, The First Affiliated Hospital of Anhui Medical University, The School of Mental Health and Psychological Sciences, Anhui Medical University, Hefei, Anhui, China; ^3^Anhui Province Key Laboratory of Cognition and Neuropsychiatric Disorders, Hefei, China; ^4^Collaborative Innovation Center of Neuropsychiatric Disorders and Mental Health, Hefei, Anhui, China; ^5^Institute of Artificial Intelligence, Hefei Comprehensive National Science Center, Hefei, China

**Keywords:** Alzheimer’s disease, cognitive function, optical coherence tomography angiography, retinal structure, vascular density, neuroimaging, MRI

## Abstract

**Introduction:**

Pathological changes in Alzheimer’s disease can cause retina and optic nerve degeneration. The retinal changes are correlated with cognitive function. This study aimed to explore the relationship of retinal differences with neuroimaging in patients with Alzheimer’s disease, analyze the association of cognitive function with retinal structure and vascular density, and identify potential additional biomarkers for early diagnosis of Alzheimer’s disease.

**Method:**

We performed magnetic resonance imaging (MRI) scans and neuropsychological assessments in 28 patients with mild Alzheimer’s disease and 28 healthy controls. Retinal structure and vascular density were evaluated by optical coherence tomography angiography (OCTA). Furthermore, we analyzed the correlation between neuroimaging and OCTA parameters in patients with mild Alzheimer’s disease with adjustment for age, gender, years of education, and hypertension.

**Results:**

In patients with mild Alzheimer’s disease, OCTA-detected retinal parameters were not significantly correlated with MRI-detected neuroimaging parameters after Bonferroni correction for multiple testing. Under multivariable analysis controlled for age, gender, years of education, and hypertension, the S-Hemi (0–3) sector of macular thickness was significantly associated with Mini-cog (β = 0.583, *P* = 0.002) with Bonferroni-corrected threshold at P < 0.003.

**Conclusion:**

Our findings suggested decreased macular thickness might be associated with cognitive function in mild AD patients. However, the differences in retinal parameters didn’t correspond to MRI-detected parameters in this study. Whether OCTA can be used as a new detection method mirroring MRI for evaluating the effect of neuronal degeneration in patients with mild Alzheimer’s disease still needs to be investigated by more rigorous and larger studies in the future.

## 1. Introduction

Alzheimer’s disease (AD) is the most common type of dementia characterized by progressive cognitive impairment. It exerts a heavy social burden on patients and their families (Brookmeyer et al., 2007). The prevalence of AD is expected to double by 2050 with the aging of the population (Hebert et al., 2013). Currently, the diagnosis of AD mainly includes several comprehensive examinations, such as magnetic resonance imaging (MRI), positron emission tomography (PET), blood tests, and cognitive assessment (Reitz and Mayeux, 2014; Rabinovici et al., 2019). Cerebrospinal fluid detection may reflect pathological changes of AD characterized by decreased amyloid and increased tau protein levels, respectively (Mantzavinos and Alexiou, 2017). However, these methods are expensive, time-consuming, invasive, and may be unsuitable for early large-scale AD screening. There is a need for a simple non-invasive biomarker, given the high incidence of AD and the lack of effective therapy late in the disease.

The retina can be used as a model for examining neuronal and microvascular damage in the brain. During embryonic development, the retina, optic nerve, and midbrain develop from the extension of forebrain vesicles. They are considered as part of the central nervous system (Patton et al., 2005; London et al., 2013). There is increasing evidence that the brain and retina are affected in patients with AD (Zhang et al., 2022). Additionally, pathological AD hallmarks have been observed in the retina, including neuron loss caused by hyperphosphorylation of tau protein (p-tau), Aβ plaques, and neurofibrillary tangles (Ho et al., 2012; Cabrera DeBuc et al., 2017; Golzan et al., 2017; Koronyo et al., 2017). Retinal plaques in animal models can be detected earlier than those in the cerebral and accumulate with disease development (Koronyo-Hamaoui et al., 2011). A recent study reported a strong correlation between retinal Aβ42 and Aβ40 levels with corresponding hippocampal levels, as well as neurofibrillary tangles and Aβ scores (Schultz et al., 2020).

Previous studies have confirmed changes in retinal morphology, structure, and vascular density in early AD patients, as well as their correlation with various cognitive functions (Almeida et al., 2019; Yan et al., 2021). Thinning of the retinal nerve fiber layer (RNFL) could reflect the loss of retinal ganglion cells, which is closely associated with brain atrophy (Shi et al., 2019b). Moreover, the total retinal thickness was positively correlated with visual gray matter volume in a mouse model of AD (Chiquita et al., 2019). Thinning RNFL, ganglion cell layer, and inner plexiform layer (GC-IPL) components are widely considered as neural parameters reflecting AD progression (Zhang et al., 2021). A study on the relationship between retinal and brain morphology in elderly people without dementia reported a positive correlation of RNFL thickness with brain volume (Shi et al., 2019b). RNFL thickness may also reflect cingulate cortex atrophy in individuals without dementia (Shi et al., 2019a). Other studies have reported a correlation between retinal thickness and brain structure in patients with AD, but these findings are inconsistent (Yoon et al., 2019; Zhao et al., 2020; Sergott et al., 2021).

Additionally, vascular factors could be critically involved in AD occurrence and development (Brown and Thore, 2011; Cortes-Canteli and Iadecola, 2020). Studies have confirmed that patients with AD have reduced cerebral blood flow (CBF) before cerebral pathological changes. The CBF may be used as a marker to predict the risk of cognitive damage (Ruitenberg et al., 2005; Cortes-Canteli and Iadecola, 2020; Kim et al., 2020). Currently, no studies have addressed retinal and CBF perfusion. Thus, the present study sought to further explore the relationship between the retina and cerebral blood perfusion by examining the correlation between the retina and brain structure, which was facilitated by optical coherence tomography angiography (OCTA) that allows not only morphological analysis but also three-dimensional (3D) visualization of retinal and choroidal vascular structures without using fluorescent dyes (Wang and Miller, 2019).

Specifically, this study aimed to explore the relationship between OCTA-detected retinal parameters and MRI-detected brain imaging parameters in patients with mild AD. We hypothesized that the differences in retinal parameters would mirror the brain imaging parameter differences in mild AD patients.

## 2. Materials and methods

### 2.1. Participants

We enrolled 28 mild AD patients aged 50 years and older and 28 healthy controls matched according to sex, age, and educational years, from the Memory Disorder Clinic of the Department of Neurology, the First Affiliated Hospital of Anhui Medical University, Hefei, Anhui, China. Mild AD was diagnosed based on the criteria from the National Institute of Neurological and Communicative Diseases and Stroke/Alzheimer’s Disease and Related Disorders Association by an experienced neurologist specialized in memory disorder, combined with an MMSE score of 16–27 and a Clinical Dementia Rating (CDR) score between 0.5 and 1 (McKhann et al., 1984, 2011; Morris, 1993).

We excluded participants with a history of non-AD-associated dementia, diabetes mellitus, uncontrolled hypertension, demyelinating disorders, or other vitreoretinal pathologic features that could interfere with OCTA analysis. The following ophthalmic examinations, including corrected visual acuity, slit-lamp microscopy, indirect fundoscopy, and intraocular pressure, were taken by an experienced ophthalmologist to exclude eye diseases [e.g., eye trauma, eye surgery, age-related macular degeneration, glaucoma, significant media opacity, high refractive error (plus or minus 3D diopters) or other ocular disorders]. Furthermore, we excluded participants with other severe mental illnesses or clinical symptoms, including major depression and a history of alcohol or substance abuse. Finally, we excluded participants with dental prostheses, pacemakers, cochlear implants, and other metal implants, and those with claustrophobia and other contraindications to MRI scanning. All participants had normal or corrected hearing and vision.

All participants underwent neuropsychological evaluations and MRI scanning. Moreover, all patients with AD underwent OCTA. The patients and their families signed consents before participating in this study, according to the Declaration of Helsinki. This study was approved by the Ethics Committee of Anhui Medical University, Hefei, Anhui, China (2019H006).

### 2.2. OCTA image acquisition

We obtained non-invasive information regarding the peripapillary and macular vascular structures using a True XR OCT device (Optovue, Inc., Fremont, CA, USA) with an 840-nm diode laser source at a sweeping rate of 70,000 A-scans every second. Retinal and choroidal vascular structures were visualized using the AngioVue software (2016.2.0 version), which quantitatively measures the vascular density and auto-locates each retinal layer. Both eyes were examined for each AD subject, and the means of OCTA parameters were calculated in the statistical analysis.

**FIGURE 1:**
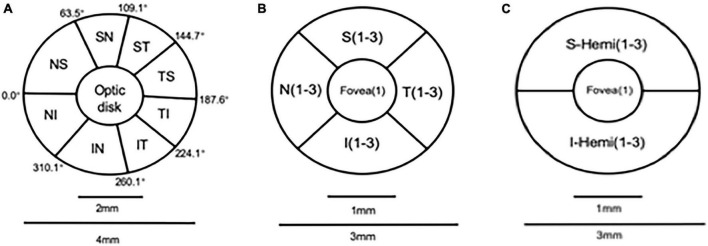
Optic disc partition mode diagram: **(A)** Garway-Heath map (eight parts); angio retina macular partition mode diagram: **(B,C)** Hemisphere and Quadrant Maps.

We obtained the peripapillary area by an area scan of 4.5 mm × 4.5 mm centered on the optic disc and macular OCTA images by an area scan of 3 mm × 3 mm centered on the fovea. The radial peripapillary capillary (RPC) layer of the peripapillary area was divided into eight sectors (superotemporal, superonasal, inferotemporal, inferonasal, nasal superior, nasal inferior, temporal superior, and temporal inferior) based on the Garway-Heath map. The RPC layer extends from the internal limiting membrane (ILM) to the posterior boundary of the RNFL (Wang et al., 2020). Additionally, we divided the macular area into two areas (superior and inferior hemispheres, S-hemi/I-hemi) or four sectors (superior, inferior, nasal, and temporal) based on the Hemisphere and Quadrant Maps (Figure 1). For measurement, the vessel density of RPC was evaluated automatically in segmented regions, including the whole image, inside disc, and peripapillary area. The whole image was evaluated over an area scan of 4.5 mm × 4.5 mm centered on the optic disc. Inside disc refers to the area inside an ellipse fitted to the optic disc boundary. The peripapillary region was measured in a 0.75-mm-wide elliptical annulus extending outward from the optic disc boundary. Each region was divided into capillary RPC, which only contained capillary, and all RPC, including the entire vascular supply in this area. The vessel density was defined as the percentage of the peripapillary region occupied by blood vessels (Rao et al., 2016; Montorio et al., 2022). The macular vessel density was divided into the deep capillary plexus with an inner boundary at 10 mm above the inner plexiform layer (IPL) as well as an outer boundary at 10 mm beneath the outer plexiform layer (OPL) and superficial capillary plexus with an inner boundary at the ILM as well as an outer boundary at 10 mm above the IPL (Wang et al., 2020). The vessel density was defined as the percentage of the macular region occupied by blood vessels.

**FIGURE 2:**
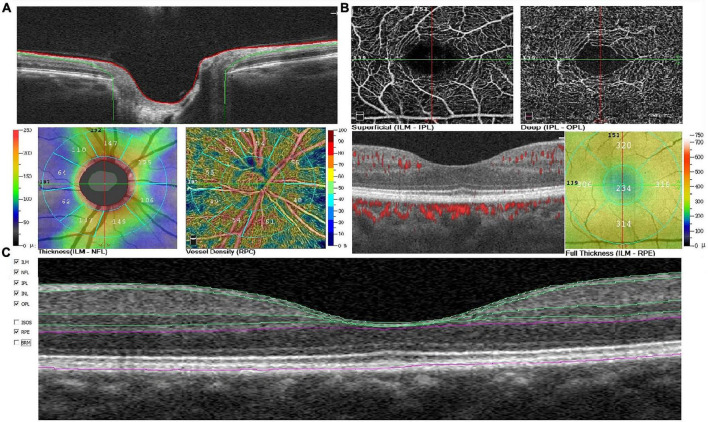
Example of optic disc **(A)**, macular **(B)** changes, and macular sublayers **(C)** automatically segmented by the AngioVue software in a patient with mild AD. AD, Alzheimer’s disease; RPC, radial parapapillary capillary; ILM, internal limiting membrane; NFL, nerve fiber layer; IPL, inner plexiform layer; INL, inner nuclear layer; OPL, outer plexiform layer; RPE, retinal pigment epithelium.

Further, we measured the thickness of peripapillary area from ILM to RNFL and total macular thickness from ILM to retinal pigment epithelium (RPE) in the areas above (Figure 2).

Finally, the operator evaluated the en face image quality and performed rescanning in case of excessive motion not correctable on the software after completing the scan. We examined the image quality and deleted images with motion artifacts or blackline images resulting from sudden eye movements (quality index < 4/10).

### 2.3. MRI acquisition

All participants were scanned on a 3.0 T magnetic resonance scanner equipped using a 32-channel phased-front coil (Discovery MR 750, GE Healthcare, Milwaukee, USA) in the Magnetic Resonance Room of the First Affiliated Hospital of Anhui Medical University. All the participants performed 3D T1-weighted imaging acquisition (repetition time/echo time = 8.5 ms/3.2 ms, TI = 450 ms, reversal angle = 15^°^, field of view = 256 mm × 256 mm, matrix = 256 mm × 256 mm, slice thickness = 1 mm, voxel size = 1 mm × 1 mm × 1 mm, scanning time ≈ 4 min, a total of 188 sagittal position images were scanned) and three-dimensional pseudo-continuous artery spin labeling (3D-pCASL) acquisition (repetition time/echo time = 5357 ms/10.7 ms, matrix = 240 mm × 240 mm, delay time = 2500 ms, NEX = 3 times, field of view = 240 mm × 240 mm, matrix = 512 dots × 8 arms, slice thickness = 4 mm) (Li et al., 2020). Before the scan, all participants rested for approximately 15 min, and metals and other contraindicated items for the MRI scan were removed from the body. Additionally, the patients were asked to close their eyes during the scan, stay awake throughout the scan, and attempt to remain still. The participants were asked to lift their legs as a signal to stop the scan when they felt uncomfortable. During the scanning process, all participants used earplugs to reduce scanning noise, and foam pads were placed to fill gaps around the coils to reduce the patient’s head movement.

### 2.4. MRI analysis

The 3D T1 data were pre-processed using the SPM8 software package^1^ using MATLAB (MathWorks, Natick, MA, USA). We use SPM12 software to implement a new segmentation algorithm that divides the T1-weighted structure image into white matter, gray matter, and cerebrospinal fluid (CSF). Affine regularization applied the International Consortium of Brain Maps template to deal with the brain of European patients, and the rest used default parameters. Next, using the Deformed Anatomy Registration by Exponential Lie Algebra (DARTEL) registration process implemented by SPM8, we normalized the six tissue probabilistic map spaces to the Montreal Institute of Neurology space to obtain 1.5 mm^3^ × 1.5 mm^3^ × 1.5 mm^3^ voxels. Whereafter, it was smoothed by convolution using an isotropic Gaussian kernel of 8-mm full width at half maximum to generate the final gray matter volume map. By combining the gray and white matter images generated during the SPM segmentation process, the total brain volume (TBV) was calculated. We calculated the intracranial volume by adding the segmented CSF volume to the TBV. Further, we calculated the ASL data using the AW4.6 GE workstation to calculate the CBF map. Using the SPM8 software package applied in MATLAB, we initially registered the CBF image of each participant to their T1 image, normalized it to the standard brain, and lastly adopted an 8-mm isotropic Gaussian kernel for smoothing (Takano et al., 2020).

### 2.5. Neuropsychological assessments

All neuropsychological assessments were conducted by senior neuropsychology graduate students in the neurology department 1 week before and after collecting the OCTA and MRI data. Specifically, the following series of neuropsychological tests were applied for the comprehensive assessment of cognitive function and clinical symptoms (Kessels and Hendriks, 2016): Mini-Cog, Mini-Mental State Examination (MMSE), Montreal Cognitive Assessment, Hachinski Ischemic Scale, Lawton-Brody Activities of Daily Living Scale, Clinical Dementia Rating, Hamilton Depression Scale (HAMD), and Hamilton Anxiety Scale (HAMA). Additionally, the following tests were used to assess individual cognition domains: Chinese version of the auditory verbal learning test (immediate, delay, and recall), Stroop Color and Word Test (dot, word, and colored word), Digital Span Test (forward/backward), Verbal Fluency Test (letter and semantic), Trail Making Test A/B, and Clock-Drawing Test.

### 2.6. Statistical analysis

All statistical analyses were performed using SPSS for MAC (23.0, IBM). The independent sample *t*-test and the Mann-Whitney *U* test were used to compare the normal distribution and non-normal distribution measurement dataset between groups. Chi-square test was used to analyze the sex differences between the groups. Partial Spearman’s rank correlation, covaried for age, gender, years of education, and hypertension, was used to assess the relationship between OCTA parameters, cerebral blood perfusion, and MRI-detected brain volume. Bonferroni correction was used for multiple testing. We then conducted a multivariable linear regression analysis of variables with *P* value < 0.05 in partial Spearman correlations, adjusting for age, gender, education, and hypertension. The Bonferroni-corrected significance was determined at *P* < 0.003. All reported beta coefficients (β) are standardized β. In the SPM8 software analysis, the whole brain volume was used as a covariable. Further, brain regions with significant differences in voxel-based morphometry (VBM) and CBF (Family wise error correction at the cluster level) were considered as regions of interest (ROI). Data Processing & Analysis for Brain Imaging (DPABI) software package was used to extract the VBM and CBF values in the ROIs for inter-group comparisons of the VBM and CBF data, with the significance level set at *P* < 0.05.

## 3. Results

### 3.1. Demographic characteristics

A total of 56 participants and 56 eyes from 28 mild AD patients were enrolled in this study. Two eyes from two participants were excluded due to poor OCTA image quality, and the other eyes from the same patients were excluded accordingly. There were no significant differences in terms of sex (χ*^2^* = 0.074, *P* = 0.786), age (64.96 ± 10.16 vs. 64.85 ± 8.56, *T* = 0.044, *P* = 0.965), years of education (8.18 ± 4.87 vs. 10.63 ± 5.26, *T* = −1.794, *P* = 0.079), and demographic characteristics between the AD (12 male, 16 female) and control (11 male, 17 female) groups. Further, the AD group showed poorer cognitive function and clinical symptoms than the control group. There was no significant inter-group difference in the HAMA and HAMD scores (Table 1).

**TABLE 1:** Neuropsychological tests of AD and HCs (x ± s).

	AD (n = 28)	HC (n = 28)	*T/Z* value	*P* value
**Clinical symptom measures**				
Mini-mental state examination^c^	21.64 ± 5.21	28.37 ± 1.47	−***5.696***	***P* < *0.001***
Montreal cognitive assessment^b^	15.89 ± 5.93	24.96 ± 2.67	−***7.257***	***P* < *0.001***
Mini-Cog^c^	0.77 ± 0.95	2.37 ± 0.79	−***4.937***	***P* < *0.001***
ADL^c^	24.64 ± 6.01	20.67 ± 2.51	−***4.273***	***P* < *0.001***
Clinical dementia rating^c^	0.77 ± 0.42	0.08 ± 0.19	−***6.033***	***P* < *0.001***
Hamilton anxiety rating scale^c^	5.79 ± 5.32	5.30 ± 4.69	−0.102	0.919
Hamilton depression rating scale^c^	4.25 ± 4.90	3.59 ± 4.64	−0.825	0.409
**Multi-domain cognition assessments**				
**Memory function assessment**				
CAVLT-immediate^b^	4.25 ± 1.94	8.57 ± 1.70	−***8.704***	***P* < *0.001***
CAVLT-delay^b^	2.85 ± 3.09	9.26 ± 2.71	−***8.106***	***P* < *0.001***
CAVLT-recognition^c^	12.30 ± 1.98	14.19 ± 0.92	−***4.281***	***P* < *0.001***
**Attention function assessment**				
Digital span test-Forward^c^	6.14 ± 1.41	7.04 ± 1.45	−***2.199***	***0.028***
Digital span test-backward^c^	3.50 ± 1.17	4.15 ± 1.20	−***2.017***	***0.044***
**Executive function assessment**				
SCWT-dot(s)^b^	36.47 ± 24.23	16.92 ± 4.57	−***8.106***	***P* < *0.001***
SCWT-word(s)^c^	48.48 ± 38.25	21.49 ± 6.39	−***4.475***	***P* < *0.001***
SCWT-color word (s)^b^	59.06 ± 58.31	34.33 ± 11.48	***2.122***	***0.043***
Color trails test - I(s)^c^	120.81 ± 8.00	74.66 ± 35.45	−***3.051***	***0.002***
Color trails test - II(s)^c^	216.16 ± 127.75	167.19 ± 182.28	−***2.741***	***0.006***
**Language function assessment**				
Verbal fluency test-letter^b^	12.57 ± 4.10	18.48 ± 3.94	−***4.274***	***P* < *0.001***
Verbal fluency test-sematic^b^	4.39 ± 2.74	7.44 ± 2.55	−***5.453***	***P* < *0.001***
**Visual-spatial function assessment**				
Clock drawing test^c^	2.31 ± 1.54	3.22 ± 1.22	−***2.214***	***0.027***

^b^Independent sample t-test; ^c^Mann–Whitney U test. AD, Alzheimer’s disease; HCs, healthy controls; ADL, Lawton-Brody Activities of Daily Living scale; CAVLT, Chinese version of the auditory verbal learning test; SCWT, Stroop Color Word Test. The bold values indicate P < 0.05.

### 3.2. Neuroimaging differences

**FIGURE 3:**
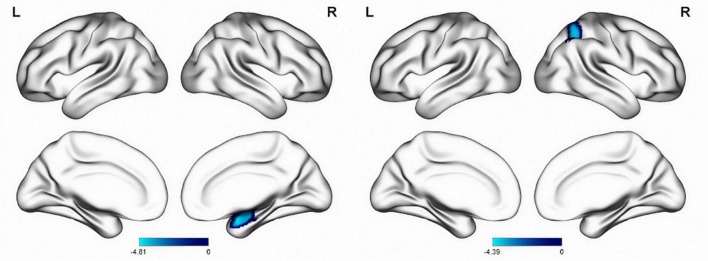
Inter-group differences in VBM **(left)** and CBF **(right)** values. The blue area of the color bar represents brain areas with decreased VBM and CBF values. VBM, voxel-based morphometry; CBF, cerebral blood flow.

Independent sample t-test analysis using SPM12 software revealed significant inter-group differences in the VBM values within clusters dominated by the right hippocampus and right amygdala, as well as CBF values mainly in the angular gyrus and right parietal lobe (all *P* < 0.05) (Figure 3 and Table 2).

**TABLE 2:** Difference of VBM and CBF values between AD and HCs.

Anatomical location	MNI coordinate X, Y, Z	Cluster size	*T* Value	*P* Value
**VBM value**				
Right hippocampus and right amygdala	33, −6, −21	1316	4.81	**0.004**
**CBF value**				
Angular gyrus and right parietal lobe	40, −60, 42	546	4.39	**0.012**

Voxel = 2 × 2 × 2 mm^3^; MNI coordinate: MNI coordinates corresponding to the voxel where the *t*-value peak in the cluster is located. AD, Alzheimer’s disease; HCs, healthy controls; MNI, Montreal Neurological Institute; VBM, voxel-based morphometry; CBF, cerebral blood perfusion. The bold values indicate *P* < 0.05.

### 3.3. Correlation between retinal parameters and MRI measurements

We analyzed the correlations between OCTA parameters and neuroimaging data using partial Spearman’s rank correlation coefficients, with adjustment for age, gender, years of education, and hypertension. In the AD group, the gray matter volume was positively correlated with temporal (1–3) (*r* = 0.436, *P* = 0.033), superior (1–3) (*r* = *0.438, P* = *0.032*), nasal (1–3) (*r* = *0.44, P* = *0.031*), inferior (1–3) (*r* = *0.424, P* = *0.039*), S-Hemi (1–3) (*r* = *0.445, P* = *0.029*), I-Hemi (1–3) (*r* = *0.432, P* = *0.035*), S-Hemi (0–3) (*r* = *0.453, P* = *0.026*) and I-Hemi (0–3) (*r* = *0.408, P* = *0.048*) sector of the macular thickness (ILM-RPE), respectively (Table 3). The vessel density of the superficial capillary plexus in the fovea (*r* = −*0.547, P* = *0.043*) and GH-SN RPC (*r* = *0.525, P* = *0.012*) were correlated with CBF, respectively. Moreover, the vessel density of capillary RPC (*r* = *0.641, P* = *0.001*) as well as all RPC (*r* = *0.554, P* = *0.006*) inside the optic disc was found to be positively associated with gray matter volume, respectively (Tables 3, 4). However, all the above associations were not statistically significant after Bonferroni correction for multiple testing (Bonferroni-corrected threshold at *P* < 0.0004). Under multivariable analysis, the S-Hemi (0–3) sector of the macular thickness (β = 0.289, *P* = 0.022) and the vessel density of capillary RPC (β = 0.813, *P* = 0.010) inside the optic disc was significantly associated with gray matter volume, respectively (Table 5). Nevertheless, none of these associations were statistically significant after Bonferroni correction (Bonferroni-corrected threshold at *P* < 0.003). Unexpectedly, the retinal thickness in the peripapillary area and the macular vascular density were not significantly correlated with neuroimaging parameters in our study (Tables 3, 4).

**TABLE 3:** Partial Spearman correlation analysis of neuroimaging and macular parameters in mild AD group with adjustment for age, gender, education, and hypertension.

		CBF_ROI	VBM_ROI	TBV	GMV	WMV
**Thickness (μm)**						
Fovea (1)	*r*	0.219	−0.075	0.239	0.303	0.363
	*P* Value	0.303	0.727	0.26	0.15	0.081
Temporal (1–3)	*r*	0.35	−0.027	0.206	0.436	0.299
	*P* Value	0.093	0.899	0.334	0.033*	0.156
Superior (1–3)	*r*	0.352	0.008	0.091	0.438	0.158
	*P* Value	0.092	0.972	0.671	0.032*	0.462
Nasal (1–3)	*r*	0.377	−0.117	0.1	0.44	0.181
	*P* Value	0.07	0.587	0.641	0.031*	0.397
Inferior (1–3)	*r*	0.314	−0.11	0.169	0.424	0.205
	*P* Value	0.135	0.61	0.43	0.039*	0.336
S-Hemi (1–3)	*r*	0.298	−0.024	0.194	0.445	0.254
	*P* Value	0.157	0.912	0.363	0.029*	0.231
I-Hemi (1–3)	*r*	0.329	−0.12	0.175	0.432	0.223
	*P* Value	0.116	0.577	0.414	0.035*	0.296
S-Hemi (0–3)	*r*	0.352	−0.074	0.202	0.453	0.281
	*P* Value	0.092	0.732	0.344	0.026*	0.183
I-Hemi (0–3)	*r*	0.355	−0.106	0.148	0.408	0.23
	*P* Value	0.089	0.623	0.491	0.048*	0.279
**Vessel density of superficial capillary plexus (%)**						
Fovea	*r*	−0.547	0.19	0.044	−0.018	0.353
	*P* Value	**0.043** *	0.514	0.88	0.951	0.216
Para fovea	*r*	0.052	0.242	0.06	0.125	0.124
	*P* Value	0.811	0.254	0.781	0.561	0.563
Temporal	*r*	0.051	0.235	0.009	0.081	0.059
	*P* Value	0.813	0.27	0.968	0.707	0.783
Superior	*r*	0.077	0.24	0.06	0.182	0.043
	*P* Value	0.722	0.259	0.782	0.395	0.842
Nasal	*r*	0.088	0.285	0.004	0.086	0.122
	*P* Value	0.683	0.178	0.984	0.689	0.571
Inferior	*r*	−0.02	0.218	0.182	−0.003	0.262
	*P* Value	0.927	0.306	0.396	0.989	0.217
**Vessel density of deep capillary plexus (%)**						
Fovea	*r*	−0.074	0.032	0.131	0.031	0.294
	*P* Value	0.73	0.88	0.543	0.886	0.163
Para fovea	*r*	−0.003	−0.063	0.164	0.205	0.129
	*P* Value	0.99	0.771	0.444	0.338	0.549
Temporal	*r*	−0.073	−0.053	−0.039	0.051	−0.046
	*P* Value	0.735	0.807	0.857	0.814	0.832
Superior	*r*	0.094	−0.18	0.176	0.251	0.111
	*P* Value	0.661	0.399	0.412	0.237	0.606
Nasal	*r*	0.068	0.013	0.148	0.178	0.178
	*P* Value	0.754	0.951	0.491	0.405	0.406
Inferior	*r*	0.023	−0.166	0.258	0.211	0.219
	*P* Value	0.916	0.438	0.223	0.322	0.303

Partitioning according to the Hemisphere and Quadrant Maps; CBF_ROI: Brain regions with significant differences in CBF values (angular gyrus, right parietal lobe); VBM_ROI: Brain regions with significantly different VBM values (right hippocampus, right amygdala). The bold values indicate *P < 0.05. The Bonferroni threshold for significance was *P* < 0.0004. TBV, total brain volume; GMV, gray matter volume; WMV, white matter volume; S-Hemi, superior hemispheres; I-Hemi, inferior hemispheres.

**TABLE 4:** Partial correlation analysis of neuroimaging and peripapillary parameters in mild AD group with adjustment for age, gender, education, and hypertension.

		CBF_ROI	VBM_ROI	TBV	GMV	WMV
**Thickness (μm)**						
Peripapillary	*r*	0.077	0.337	0.169	0.08	0.32
	*P* Value	0.732	0.125	0.452	0.722	0.146
GH-TS	*r*	0.372	−0.019	−0.046	0.346	0.095
	*P* Value	0.081	0.93	0.835	0.106	0.667
GH-ST	*r*	0.176	0.288	−0.038	−0.011	0.061
	*P* Value	0.433	0.194	0.867	0.96	0.789
GH-SN	*r*	−0.045	0.091	0.176	−0.144	0.012
	*P* Value	0.843	0.686	0.433	0.522	0.957
GH-NS	*r*	−0.278	0.053	0.158	−0.321	0.373
	*P* Value	0.198	0.811	0.471	0.135	0.079
GH-NI	*r*	−0.387	0.11	0.026	−0.219	0.209
	*P* Value	0.068	0.617	0.905	0.315	0.338
GH-IN	*r*	−0.125	0.079	0.084	−0.244	0.191
	*P* Value	0.569	0.721	0.704	0.261	0.384
GH-IT	*r*	0.104	0.368	−0.073	0.011	0.029
	*P* Value	0.636	0.084	0.741	0.959	0.897
GH-TI	*r*	0.232	0.251	−0.242	0.151	−0.109
	*P* Value	0.287	0.247	0.267	0.492	0.621
**Vessel density (%)**						
**Capillary RPC**						
Peripapillary-Capillary_RPC	*r*	0.389	0.172	0.006	0.177	0.019
	*P* Value	0.073	0.445	0.978	0.431	0.933
Inside Disc-Capillary_RPC	*r*	0.336	0.11	0.022	0.641	−0.1
	*P* Value	0.117	0.617	0.919	0.001**	0.65
Whole Image Capillary_RPC	*r*	0.258	0.095	−0.178	−0.113	−0.136
	*P* Value	0.234	0.665	0.416	0.607	0.537
**All RPC**						
Pepipapillary_RPC	*r*	0.336	0.167	−0.041	0.124	0.05
	*P* Value	0.126	0.457	0.857	0.582	0.825
GH-NS_RPC	*r*	0.318	0.216	−0.043	0.043	−0.027
	*P* Value	0.139	0.323	0.845	0.847	0.902
GH-NI_RPC	*r*	0.193	0.126	−0.267	−0.266	−0.136
	*P* Value	0.379	0.568	0.218	0.22	0.537
GH-IN_RPC	*r*	0.247	0.112	−0.135	−0.051	−0.136
	*P* Value	0.256	0.612	0.538	0.816	0.536
GH-IT_RPC	*r*	0.242	0.123	−0.054	0.168	−0.119
	*P* Value	0.265	0.575	0.807	0.442	0.588
GH-TI_RPC	*r*	0.18	0.209	−0.37	−0.163	−0.182
	*P* Value	0.411	0.339	0.082	0.457	0.407
GH-TS_RPC	*r*	0.357	0.169	−0.278	0.003	−0.073
	*P* Value	0.094	0.441	0.199	0.988	0.741
GH-ST_RPC	*r*	0.162	0.138	0.277	0.127	0.119
	*P* Value	0.471	0.541	0.212	0.572	0.599
GH-SN_RPC	*r*	0.525	0.388	−0.059	0.259	−0.156
	*P* Value	0.012*	0.074	0.796	0.244	0.489
Inside Disk_RPC	*r*	0.4	0.076	0.022	0.554	−0.095
	*P* Value	0.059	0.73	0.919	0.006**	0.665
Whole Image_RPC	*r*	0.241	0.101	−0.229	−0.158	−0.115
	*P* Value	0.268	0.648	0.292	0.471	0.602

Partitioning according to the Garway-Heath map; CBF_ROI: Brain regions with significant differences in CBF values (angular gyrus, right parietal lobe); VBM_ROI: Brain regions with significantly different VBM values (right hippocampus, right amygdala). The bold values indicate *P < 0.05 and **P < 0.01. The Bonferroni threshold for significance was *P* < 0.0004. TBV, total brain volume; GMV, gray matter volume; WMV, white matter volume; GH, Garway-Heath map; RPC, radial peripapillary capillary; ST, superotemporal; SN, superonasal; IT, inferotemporal; IN, inferonasal; NS, nasal superior; NI, nasal inferior; TS, temporal superior; TI, temporal inferior.

**TABLE 5:** Multivariable analysis of retinal parameters, neuroimaging parameters, and cognitive function in mild AD group with adjustment for age, gender, education, and hypertension.

			Macular thickness							
		Inside Disk-Capillary_RPC	S-Hemi (0–3)	I-Hemi (0–3)	Temporal (1–3)	Superior (1–3)	Nasal (1–3)	Inferior (1–3)	S-Hemi (1–3)	I-Hemi (1–3)
GMV	β	***0.813***	***0.289***	−0.452	−0.377	−0.285	−0.283	−0.285	−0.382	−0.556
	*P* value	*0.010* *	***0.022*** *	0.451	0.548	0.683	0.711	0.517	0.758	0.344
Mini-Cog	β		***0.583***	0.053	**SCWT-word /**	ß −2.195/−2.193			1.891/1.926	
	*P* value		***0.002*** **	0.948	**SCWT-color word**	*P* value 0.254/0.297			0.322/0.357	

The bold values indicate *P value significant at < 0.05. **P value significant at < 0.01. The Bonferroni threshold for significance was P < 0.003. GMV, gray matter volume; RPC, radial peripapillary capillary; S-Hemi, superior hemispheres; I-Hemi, inferior hemispheres.

### 3.4. Correlation between macular thickness and cognitive function

Based on the findings, we conducted partial Spearman’s rank correlation to analyze the relationship between macular thickness and cognitive function in the AD group, covaried for age, gender, years of education, and hypertension. Interestingly, the S-Hemi (0–3) (*r* = 0.42, *P* = 0.046) and I-Hemi (0–3) (*r* = 0.425, *P* = 0.043) subfields of macular thickness were both positively correlated with the Mini-Cog score (Table 6). Although both of them were not statistically significant after correction for multiple testing (Bonferroni-corrected threshold at P < 0.0003), the S-Hemi (0–3) sector of macular thickness was found to be the independent variable associated with Mini-Cog score under multivariable analysis (β = 0.583, *P* = 0.002) with Bonferroni-corrected threshold at P < 0.003 (Table 5). In addition, the score of SCWT-Word (*r* = −*0.441, P* = *0.035*) (*r* = −*0.432, P* = *0.04*) and SCWT-Color Word (*r* = −*0.465, P* = *0.029*) (*r* = −*0.434, P* = *0.044*) tests were both associated with superior (1–3) and S-Hemi (1–3) sectors of macular thickness, but these associations became non-significant after correction for multiple comparisons and multivariable analysis (β = −2.195, *P* = 0.254; β = 1.891, *P* = 0.322; β = −2.193, *P* = 0.297; β = 1.926, *P* = 0.357, respectively) (Tables 5, 6).

**TABLE 6:** Partial Spearman correlation analysis of cognitive function and macular thickness in mild AD group with adjustment for age, gender, education, and hypertension.

		Fovea (1)	Temporal (1–3)	Superior (1–3)	Nasal (1–3)	Inferior (1–3)	S-Hemi (1–3)	I-Hemi (1–3)	S-Hemi (0–3)	I-Hemi (0–3)
Mini-Cog	*r*	0.335	0.41	0.369	0.383	0.381	0.372	0.381	0.42	0.425
	*P* value	0.118	0.052	0.083	0.071	0.073	0.08	0.072	0.046*	0.043*
MMSE	*r*	0.355	0.363	0.376	0.276	0.281	0.336	0.308	0.374	0.333
	*P* value	0.089	0.081	0.07	0.192	0.183	0.109	0.143	0.072	0.112
MoCA	*r*	0.149	0.184	0.261	0.147	0.098	0.154	0.119	0.2	0.131
	*P* value	0.488	0.388	0.218	0.493	0.65	0.473	0.581	0.349	0.542
**Multi-domain cognition assessments**										
**Memory function assessment**										
CAVLT-immediate	*r*	0.137	0.16	0.266	0.112	0.106	0.164	0.108	0.187	0.134
	*P* value	0.534	0.465	0.219	0.61	0.629	0.456	0.624	0.392	0.542
CAVLT-delay	*r*	0.093	0.029	0.096	−0.069	−0.046	−0.003	−0.058	0.035	−0.02
	*P* value	0.672	0.894	0.662	0.754	0.837	0.989	0.793	0.874	0.927
CAVLT-recognition	*r*	0.066	0.012	0.181	0.033	0.056	0.072	0.043	0.101	0.043
	*P* value	0.766	0.958	0.409	0.882	0.799	0.744	0.847	0.645	0.847
**Attention function assessment**										
DST-forward	*r*	0.003	−0.191	−0.052	−0.195	−0.193	−0.09	−0.21	−0.097	−0.193
	*P* value	0.989	0.372	0.808	0.36	0.366	0.676	0.324	0.653	0.365
DST-backward	*r*	−0.073	0.006	0.168	0.057	0.028	0.121	0.034	0.062	0.018
	*P* value	0.734	0.976	0.433	0.793	0.897	0.574	0.873	0.773	0.935
**Executive function assessment**										
SCWT-dot	*r*	−0.249	−0.265	−0.297	−0.242	−0.178	−0.273	−0.207	−0.294	−0.241
	*P* value	0.241	0.211	0.159	0.254	0.405	0.197	0.331	0.163	0.257
SCWT-word	*r*	−0.171	−0.395	−0.441	−0.321	−0.341	−0.432	−0.347	−0.409	−0.345
	*P* value	0.434	0.062	**0.035** *	0.135	0.112	0.04*	0.105	0.053	0.107
SCWT-color word	*r*	−0.223	−0.417	−0.465	−0.345	−0.375	−0.434	−0.379	−0.417	−0.381
	*P* value	0.319	0.053	0.029*	0.116	0.085	0.044*	0.082	0.054	0.08
Color trails test - I	*r*	0.129	−0.041	−0.17	−0.049	−0.035	−0.012	−0.001	−0.012	−0.02
	*P* value	0.66	0.888	0.561	0.867	0.905	0.968	0.996	0.967	0.946
Color trails test - II	*r*	0.461	0.264	0.159	0.289	0.184	0.227	0.242	0.267	0.241
	*P* value	0.113	0.383	0.604	0.338	0.548	0.456	0.426	0.377	0.428
**Language function assessment**										
VFT-letter	*r*	0.127	0.061	0.143	0.048	0.012	0.041	−0.017	0.094	0.056
	*P* value	0.555	0.778	0.506	0.825	0.956	0.848	0.936	0.663	0.795
VFT-semantic	*r*	0.109	0.26	0.231	0.166	0.195	0.237	0.164	0.194	0.182
	*P* value	0.613	0.22	0.278	0.437	0.361	0.266	0.443	0.365	0.396
**Visual-spatial function assessment**										
CDT	*r*	−0.015	0.265	0.396	0.248	0.256	0.304	0.245	0.263	0.254
	*P* value	0.946	0.222	0.061	0.253	0.239	0.158	0.259	0.226	0.243

The bold values indicate *P < 0.05. The Bonferroni threshold for significance was *P* < 0.0003. S-Hemi, superior hemispheres; I-Hemi, inferior hemispheres; MMSE, Mini-Mental State Examination; MoCA, Montreal Cognitive Assessment; CAVLT, Chinese version of the auditory verbal learning test; DST, Digital Span Test; SCWT, Stroop Color Word Test; VFT, Verbal Fluency Test; CDT, Clock-Drawing Test.

## 4. Discussion

This cross-sectional study explored the correlation of retinal neurodegeneration and brain degeneration in patients with Alzheimer ’s disease, showing that all the subfields of macular thickness except the fovea were associated with the gray matter volume. However, the associations were not significant after Bonferroni correction for multiple testing. Under multivariable analysis, the S-Hemi (0–3) sector of macular thickness and the vessel density of inside-disc capillary RPC were independently associated with gray matter volume, including confounders. Similarly, these associations were not statistically significant after Bonferroni correction. It suggests that reduced macular thickness, which reflected macular neurodegeneration in patients with AD, might correspond to pathological brain degeneration (Tao et al., 2019). Further, we found that the Mini-Cog score was related to the S-Hemi (0–3) sector of macular thickness under multivariable analysis. It provides a potential new idea for evaluating the effect of neuronal injury in patients with mild AD, as OCTA is a cost-efficient, non-invasive, and easily acquired test compared to cerebrospinal fluid or PET examination (Czakó et al., 2020).

Our findings showed that there might be a positive correlation between gray matter volume and macular thickness in patients with AD. Thinner macular thickness indicates a reduced number of retinal neurons in the entire layer of the macula in Alzheimer’s disease (Cheung et al., 2019). Retinal neural degeneration has been discovered in AD patients and transgenic mice (Zhang et al., 2022). Interestingly, a longitudinal multimodal *in vivo* study showed a positive correlation between total retinal thickness and visual cortex gray matter volume in a mouse model of AD (Chiquita et al., 2019). This may suggest a possible developmental link between the brain and retinal volume, with the association of neurodegenerative changes to the concurrent loss of retinal thickness. Similarly, Haan et al. reported that total macular thickness is correlated to parietal cortical atrophy both in early onset AD patients and healthy controls, which may suggest a reflection of cerebral cortical changes in the retina, independent of amyloid (den Haan et al., 2018). Another research reported that TBV was positively correlated with the volume from the macular ellipsoid to the retinal pigment epithelium among healthy elderly people, which may indicate the developmental connection between the brain and retinal tissue volume (Uchida et al., 2020). Notably, this association has been confirmed in several neurodegenerative diseases. Recent studies have reported that RNFL thickness is also associated with brain atrophy in multiple sclerosis, AD, and other neurodegenerative diseases (Frau et al., 2018; Vural et al., 2018; Zhao et al., 2020).

Moreover, we analyzed the correlation between retinal parameters and cognitive function, which were associated with grey matter volume. Unexpectedly, we found that the S-Hemi (0–3) and I-Hemi (0–3) sectors of macular thickness were both correlated with the Mini-Cog rather than MMSE. Although they became non-significant after Bonferroni correction of multiple testing, the S-Hemi (0–3) sector of macular thickness remained significantly associated with Mini-Cog under multivariable analysis even after Bonferroni correction. The Mini-Cog is a short cognitive test comprising three recall items and a Clock-Drawing Test, which is used in secondary healthcare settings as a screening tool for patients requiring further professional diagnosis and evaluation (Js et al., 2000; Chan et al., 2019). The MMSE assesses the overall cognitive function and has a higher sensitivity in identifying patients with dementia (Carpinelli Mazzi et al., 2020). Some studies demonstrated that retinal thickness was associated with MMSE score in AD patients (Kim and Kang, 2019; Mei et al., 2021), while other researchers didn’t find such associations which are consistent with our results (Cipollini et al., 2020; Zhao et al., 2020). However, Li et al. (2018) reported that the Mini-Cog was superior to MMSE in identifying Chinese outpatients with mild cognitive impairment, which was less affected by age and education level than MMSE. MMSE best classifies moderate stage of AD severity and has been criticized by some as being insensitive in distinguishing mild cognitive impairment (Devanand et al., 2008; Benoit et al., 2020). Carnero-Pardo et al. conducted a study including 581 individuals and found that the Mini-Cog showed greater diagnostic usefulness than the CDT in detecting cognitive impairment (Carnero-Pardo et al., 2022). This may suggest that macular thickness, which might be associated with Mini-cog in our study, may play a predictive role in early AD screening (Tao et al., 2019). Furthermore, reduced macular thickness in AD patients may correspond to pathological cerebral injury (den Haan et al., 2018).

Notably, we found that only the S-Hemi (0–3) sector of macular thickness was significantly associated with Mini-cog under multivariable analysis after Bonferroni correction. Of interest, the superior macula was reported to be thicker than the inferior macula as determined by OCT in normal eyes (Huynh et al., 2006; Huang et al., 2009). In addition, the blood flow in the superior retinal peripapillary area was higher than that of the inferior retina, independent of posture in healthy people (Tomita et al., 2020). Nagatomo et al. (1998) reported that the amplitudes of multifocal electroretinograms were larger in the superior retina than in the inferior retina, suggesting the functional superiority of the superior retina. Accordingly, the greater metabolic demand of the superior retina may make it more susceptible to a hyperglycemic state than the inferior retina (Luan et al., 2004). Moreover, the density of the retinal ganglion cells was 65% higher at 4 mm superior to the fovea than at 4 mm inferior to the fovea, which may explain why the S-Hemi sector, not the I-Hemi sector of macular thickness, was significantly associated with Mini-cog under multivariable analysis in our study (Curcio and Allen, 1990).

Interestingly, we also found that the vessel density of capillary RPC inside the optic disc might be associated with gray matter volume. Similarly, the association didn’t reach statistical significance after Bonferroni correction. To our knowledge, no studies have confirmed the relationship between radial peripapillary vessel density and grey matter volume. Yoon et al. (2019) focused on the macular area and found a significant correlation between reduction in the superficial capillary plexus vessel density and perfusion density on OCTA and expansion of the inferolateral ventricle in MCI and AD. However, the RPCs are a distinctive vascular network within the RNFL around the optic disc that has fewer anastomoses when compared with the superficial capillary plexus, which may make the vessels more susceptible to vascular dysfunction (Henkind, 1967). It makes RPC an attractive target for the study of AD (Song et al., 2021).

Additionally, we extracted brain regions showing significant inter-group differences in the VBM (right hippocampus, right amygdala) and CBF (right parietal lobe, angular gyrus) values, which are correlated with retinal layer thickness and blood vessel density. However, there were no significant correlations. Several studies have reported decreased macular RNFL thickness in patients with AD, significantly correlated with the hippocampal volume. There is reduced hippocampal volume in AD progression (Zhao et al., 2020; Sergott et al., 2021). The observed differences in the correlation between brain volume and retinal structure may be attributed to our ROI extraction as a cluster that included other brain areas. However, we did not perform detailed stratification of the retina. The inner and outer retinal layers may be affected by confounding factors. The inner retinal layer may be affected if the patient possesses vascular co-morbidities, such as arteriosclerosis. Alternatively, the inner retina may be intact, while the outer retina may be damaged among patients with age-related macular degeneration (Chen et al., 2017).

To our knowledge, no studies have confirmed the relationship between CBF and retinal vessel density. Although there was no significant correlation between the CBF in different brain regions and retinal parameters in our study, a recent report demonstrated that decreased CBF might cause atrophy of retinal ganglion cells and subsequent changes in retinal vessel density caused by reduced oxygen consumption (Wang et al., 2020).

Our study has several limitations, including its small sample size and the inconclusiveness of our findings. Additionally, we only analyzed total retinal thickness without segmentation into sublayers. In the vascular analysis of OCTA, we focused on vascular density, but other vascular parameters, such as the number of vascular crossings and lacunarity, were not included. Nevertheless, our findings explored the relationship between brain volume and retinal structure. Although we didn’t completely confirm that OCTA-based retinal measurements can partially track brain MRI measurements in mild AD, it highlights the need for future larger studies to examine the relationship between OCTA-detected retinal differences and MRI-detected neuroimaging differences in mild AD and MCI.

## 5. Conclusion

In conclusion, we found that macular thickness might be positively correlated with cognitive function in patients with mild AD. This demonstrates reduced macular thickness in AD patients might correspond to cerebral injury. However, the differences in retinal parameters didn’t conform to MRI-detected parameters in this study. Whether OCTA can provide a novel detection method for evaluating neuron injury, which can be detected by MRI in mild AD, still needs to be rigorously evaluated in future larger studies.

## Data availability statement

The data analyzed in this study is subject to the following licenses/restrictions: All datasets generated for this study are included in the manuscript and/or the supplementary files. Requests to access these datasets should be directed to KW, wangkai1964@126.com.

## Ethics statement

The studies involving human participants were reviewed and approved by the Ethics Committee of Anhui Medical University. The patients/participants provided their written informed consent to participate in this study.

## Author contributions

BZ, YY, and XW performed the analysis and wrote the manuscript. ZG, YW, GX, LW, SZ, and LW helped to collect the data. RL and KW designed and supervised the study. All authors contributed to the article and approved the submitted version.

## Footnotes

1
http://www.fil.ion.ucl.ac.uk/spm/
